# Transafferent nodal embolization for lymphocele treatment: case report

**DOI:** 10.31744/einstein_journal/2022RC6889

**Published:** 2022-08-10

**Authors:** Katia Pinheiro de Souza, Guilherme Moratti Gilberto, Guilherme Cayres Mariotti, Priscila Mina Falsarella, Francisco Leonardo Galastri, Fernando Korkes, Rodrigo Gobbo Garcia

**Affiliations:** 1 Hospital Israelita Albert Einstein São Paulo SP Brazil Hospital Israelita Albert Einstein, São Paulo, SP, Brazil.

**Keywords:** Embolization, therapeutic/methods, Drainage, Lymphocele

## Abstract

Lymphoceles are collections of lymphatic fluid, mainly caused by major surgical approaches. Most lymphoceles are asymptomatic and limited, but some cases may require a medical management. Among the different techniques, transafferent nodal embolization has emerged as a minimally invasive option, with low morbidity and high resolubility, although it is not widespread in the Brazilian scenario. In this study, we report a case of lymphocele drained percutaneously, with maintenance of high output and requiring transafferent nodal embolization.

## INTRODUCTION

With the advancement of medical technologies and higher survival rates in critically ill patients, there is a growing demand for techniques to manage complications inherent to major approaches, such as lymphatic changes, once so rare and now more prevalent, but no less challenging.

Histologically, lymphoceles are differentiated from cysts by being a collection of lymphatic fluid surrounded by a fibrotic wall without a true epithelial lining.^(1)^ In general, these collections are secondary to extensive surgical procedures, such as those related to treatment of neoplasms, transplants, complex vascular procedures, and advanced life support measures (*e.g.*, extracorporeal membrane oxygenation cannulation). Other causes include infectious diseases, congenital malformations, trauma, neoplasms, and cirrhosis/portal hypertension.^(2)^ Most lymphoceles are asymptomatic and limited, but some cases may present secondary infection, or a compressive effect on adjacent structures, culminating in edema due to venous compression, pain, or pyelocaliceal dilation, among other symptoms.

Conventional therapeutic strategies include dietary modification, drainage, and supportive treatment. They are used in patients with symptomatic lymphoceles, the most frequent symptom being urinary incontinence. Regarding minimally invasive techniques, the options are embolization/sclerotherapy of the collection, percutaneous afferent lymphatic vessel embolization/sclerotherapy, and transafferent nodal embolization (TNE). These techniques are employed in cases of maintenance of drainage with high output despite supportive measures.^(3)^ In this essay, we report a case of management of lymphoceles with intranodal injection with n-butyl cyanoacrylate (NBCA) by the TNE technique.

## CASE REPORT

A 46-year-old male patient, in late postoperative period after radical prostatectomy and extended pelvic lymphadenectomy for prostate adenocarcinoma, developed an abdominal pelvic collection. Initially, bilateral percutaneous drainage of the collection was performed. Laboratory evaluation of the fluid showed no infectious complications. After 12 to 13 days of drainage, the patient maintained a daily flow through the drains of more than 100mL per day.

**Figure 1:**
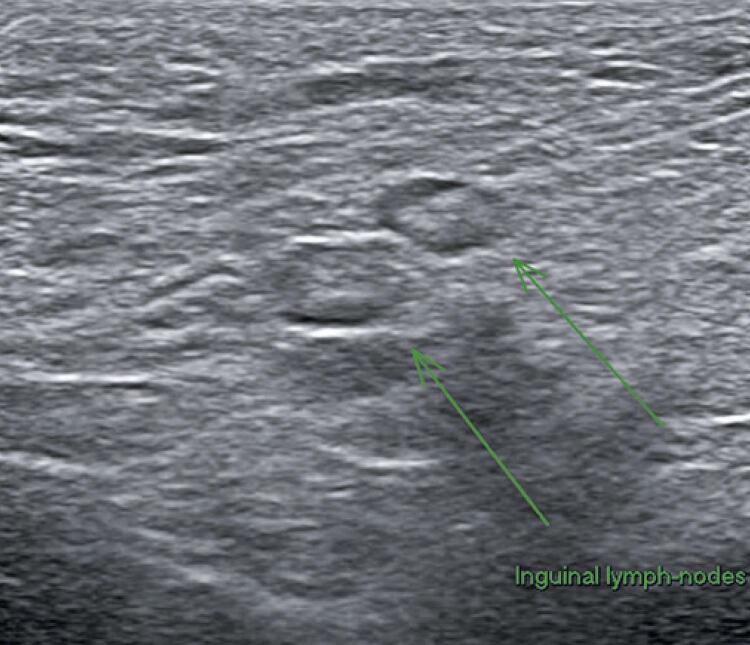
Ultrasonography demonstrating inguinal lymph nodes of a habitual size

**Figure 2:**
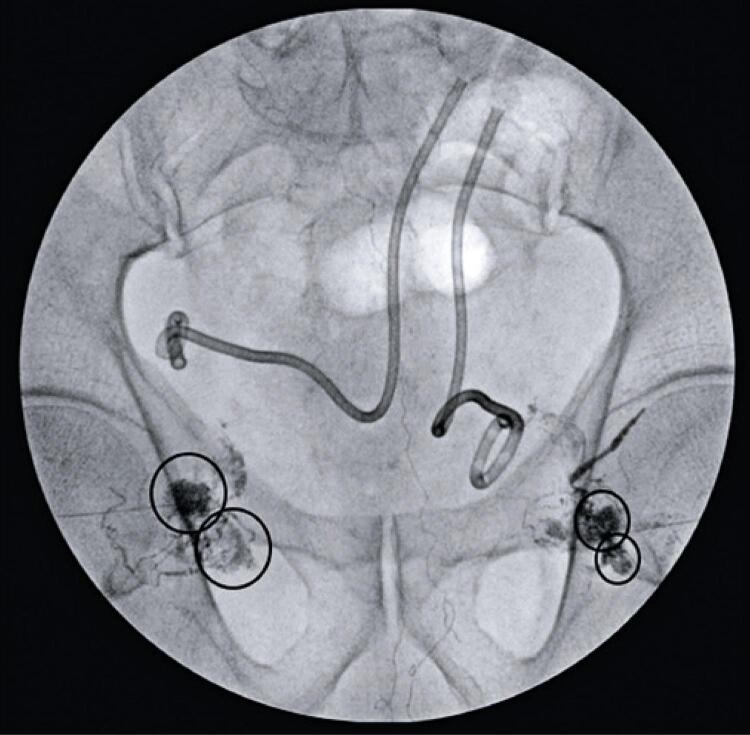
Pelvic radioscopy showing lymph nodes with enhancement (black circles) after ultrasound-guided puncture and lipiodol infusion

**Figure 3:**
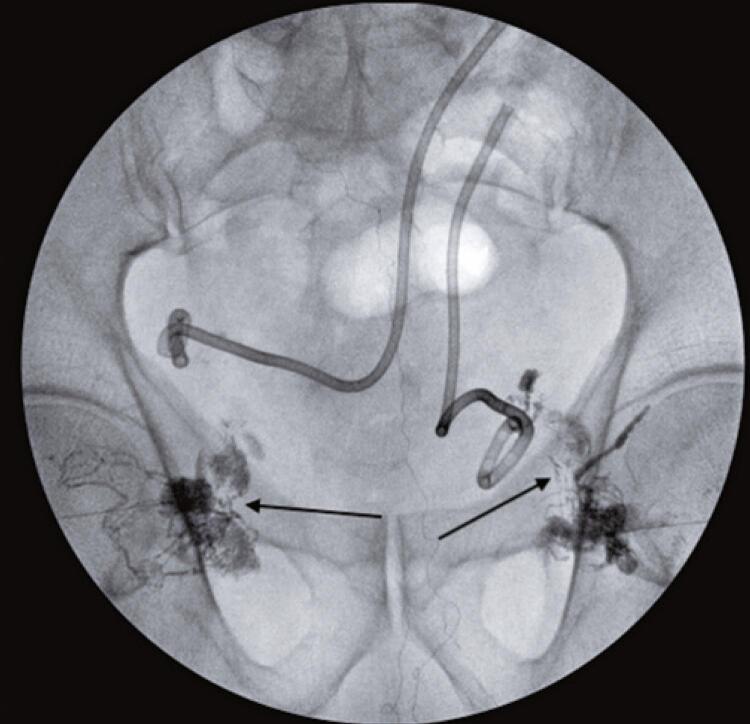
Small lymphatic vessels (black arrows) carrying the contrast to the deep pelvic basins

**Figure 4:**
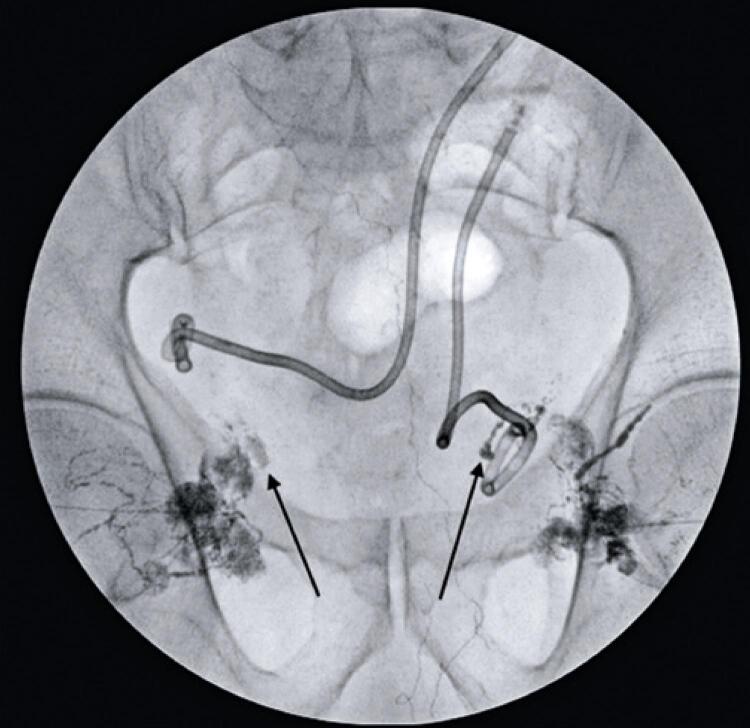
Leakage of the contrast into the lymphocele cavity (black arrows)

**Figure 5:**
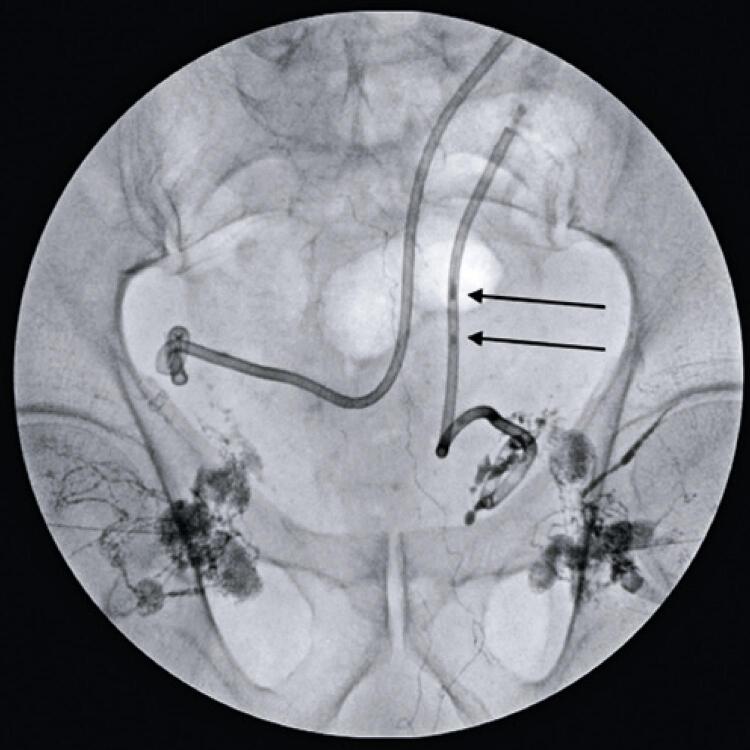
Lipiodol being aspirated through the previously allocated percutaneous drain (black arrows)

After multidisciplinary discussion, it was decided to perform TNE to close the lymphocele. Initially, an ultrasound-guided puncture of the lymph node near the fistula site was performed (Figure 1), allowing the injection of lipiodol (Figure 2) and the consequent mapping of the lymphatic system (Figure 3). This showed contrast medium leakage and identified the fistulous tract (Figure 4). Next, an intranodal injection of NBCA solution diluted with lipiodol with a ratio of 1:3 was performed. The intraprocedural control showed good diffusion of the solution through the lymphatic system, with obstruction of the fistula site (Figure 5). In the following days, a gradual reduction of the flow rates was observed, approaching zero after 7 days, thus allowing the removal of both drains. The patient continues with no new complaints.

This study was approved by the Research Ethics Committee of *Hospital Israelita Albert Einstein* under # 4.712.167, CAAE: 45766921.2.0000.0071.

## DISCUSSION

The treatment of lymphatic system disorders is a new frontier for interventional radiology. The increased availability of complex approaches to severe diseases in advanced stages, and the complications inherent to them have consequently generated a new demand for techniques in the management of lymphatic system disorders, with anatomical and functional complexity that offer an additional challenge to the interventional physician.

Lymphoceles occur in about 30% of patients after pelvic lymphadenectomy.^(4)^ A small percentage of these collections are symptomatic, requiring additional therapy. However, the recrudescence rate in lymphoceles that required the approach reaches almost 50% in some series in the literature.^(4,5)^

In this scenario, some new methods have been improved for the management of lymphatic system disorders - TNE among them. In TNE, a puncture is performed, usually guided by imaging, of a lymph node close to the region of the collection/fistula, with subsequent embolization, using glue from the lymph nodes and afferent lymphatic vessels and performing closure of the routes that directly feed the leak. This has been considered the most efficient method to treat lymphatic fistulas with multiple afferent lymphatic vessels.^(3,5)^

Regarding the technique, the ideal dilution of the liquid agent (glue) in lipiodol is not yet defined, and solutions between 1:2-1:9 have been reported.^(6,7)^ In the cases described here, a dilution of one part of glue to three parts of lipiodol was used, highlighting the good fluidity of this mixture toward the leak site. It is worth noting that treatment failures may occur when using very diluted solutions (>1:6).^(7)^ Another point to be defined is how close to the leak site the chosen lymph node should be. Such gaps should be the target of future studies.

The classical treatments for lymphoceles include surgery with open internal marsupialization and later laparoscopic or external drainage. More recently (mid-1990s), minimally invasive techniques have been used such, as suction and drainage combined or not with sclerotherapy. Percutaneous techniques have advantages over surgery, since they allow outpatient management, faster recovery, and lower costs, with similar success rates.^(1)^

Compared with sclerotherapy, TNE has been shown to be more effective in resolving lymphoceles, with clinical success rates of 80% to 100%.^(6,7)^ The additional benefit of TNE is that its efficacy does not depend on the volume initially drained. This volume is classically considered to be a function of the extent of the lesion in the lymphatic system and a limiting factor in the success of sclerotherapy.^(7)^ Moreover, TNE can be used in cavitary lymphatic collections (chylous ascites, *e.g.*,), which limit sclerotherapy, considering the risk of chemical peritonitis. Another positive point for the embolization technique would be the need for only one treatment session, considering that the injection of sclerosing agents is usually done in multiple administration protocols.

Given the embolization of multiple lymph nodes and lymphatic vessels, there is an increased risk of lymphedema as an adverse effect of the treatment. However, Kim et al.^(7)^ described the occurrence of lymphedema in only two out of 24 patients undergoing TNE. Other adverse effects described are fever and local pain, which are generally well tolerated.

## CONCLUSION

Lymphoceles can arise from several diseases or occur as complications of invasive medical procedures. The approach to these collections is by a multidisciplinary team and demands the expertise of innovative techniques. In this scenario, transafferent nodal embolization appears as a safe and effective option, and is becoming an important tool to be considered in the treatment of lymphoceles and lymphorrhea. However, further studies should be conducted to corroborate such results.
